# Cloning and in silico characterization of an abiotic stress-inducible U-box domain-containing protein gene Gs*PUB8* from *Glycine soja*

**DOI:** 10.1038/s41598-022-21583-9

**Published:** 2022-10-13

**Authors:** Ali Inayat Mallano, Zaib-un Nisa, Binish Khaliq, Naila Ali, Qurban Ali, Chen Chao, Zhu Yanming

**Affiliations:** 1 State Key Laboratory of Tea Plant Biology and Utilization, Anhui Agricultural University, West Changjiang Road, Hefei, 230036 Anhui People’s Republic of China; 2 Institute of Molecular Biology and Biotechnology, The University of Lahore, Defence Road Campus, Lahore, Pakistan; 3 Key Laboratory of Agricultural Biological Functional Genes, Northeast Agricultural University, Harbin, 150030 China; 4 Department of Botany, University of Okara, Okara, Pakistan; 5 Department of Plant Breeding and Genetics, Faculty of Agricultural Sciences, University of the Punjab, Lahore, Pakistan; 6 Department of Chemistry and Molecular Biology, School of Life Science and Technology, Harbin Normal University, Harbin, 150025 People’s Republic of China

**Keywords:** Plant biotechnology, Biotechnology

## Abstract

The ubiquitination pathway is involved in the posttranslational modification of cellular proteins. However, the role of E3 ubiquitin ligase family proteins under abiotic stress conditions remains unclear, particularly in soybean. The core objective of the current study was to isolate and functionally characterize the GsPUB8 protein gene from wild soybean (*Glycine soja*) by using a homologous cloning method to investigate its abiotic stress responses. The GsPUB8 is a 40,562 Da molecular weight protein with 373 amino acid residues. The sequence alignment revealed the presence of U-box domain while the phylogenetic analysis showed an abundance of PUB8 proteins in both monocot and dicot plants. Analysis of gene structure predicted the absence of introns along with the presence of one exon. Furthermore, the activity of the GsPUB8 protein was anticipated in the plasma membrane and its expression was persuaded with NaCl, ABA, PEG6000, and NaHCO_3_ treatments with considerably higher manifestation in roots than leaves although, expressed in both vegetative and reproductive parts of *G. soja*. GsPUB8 protein showed 54% and 32% sequence identity to U-box domain containing 8 and 12 proteins from *Arabidopsis thaliana* and *Oryza sativa* subsp. *japonica*, respectively. GsPUB8 exhibited relatively higher expression under saline and drought stress particularly in roots. Whereas, the 3D model of GsPUB8 protein was generated using the SWISS-MODEL. This study can be used to manipulate the GsPUB8 protein or *GsPUB8* gene for transformation purposes and its functional characterization under abiotic stress conditions.

## Introduction

Proteolytic enzymes, also called proteases are the well-known class of enzymes involved in breakdown of enzymes. In higher plants, a major protease (ubiquitin/26S proteasome) has been considered to be responsible for protein degradation in cytosol as well as in nucleus and is also involved in the major pathway for plant growth and development contributing to several critical cellular events including RNA metabolism, transcriptional regulation, senescence, embryogenesis and hormonal and stress signaling^1,2^. There are three main enzymes involved in the ubiquitin conjugation cascade: (i) E1, a ubiquitin activating enzyme, E2, a ubiquitin conjugating enzyme and E3 a ubiquitin ligase E3^3^. Among them, E3 ubiquitin ligases are the most abundant protein in plant systems, as these enzymes actively participate in the selection of precise target molecules for the protein degradation process. Plants possess a vast repertoire of U-box E3 ligases compared to HECT and RING domain E3 ligases^4^. Previous studies on different plant genomes have discovered multiple E3 ubiquitin ligases in model organisms such as rice and *Arabidopsis*. The cross-talk between protein degradation and regulation of plant growth and development was predicted after the apparent involvement of this vast gene family in multiple biological processes such as hormonal responses and biotic/abiotic stress tolerances^5^. A U-box domain was first identified in the yeast UFD2 protein, which belongs to the category of proteins with RING-finger domains^6,7^. Proteins of plant U-box family are further categorized into five main groups due to presence of ARM repeats, UFD2, WD40 repeats, UND and Ser/Thr kinase domains^8^.

The plant U-box genes (*PUB*) regulate hormone signal transduction of plant and also tolerate abiotic stresses including saline environment^9,10^. Intriguingly, the existence of the U-box domain in many stress-response genes implies that these genes might be helpful in activating plant adaptability under severe environmental stresses through the degradation of proteins and modulation of the plant proteomes. Nevertheless, so far, no direct evidence exists in the literature to suggest that the wild soybean PUB domain-containing family proteins provoke protein degradation and regulation of plant growth and development under salinity stress. *Glycine soja* is one of the wild species of soybean with a prominently high tolerance rates to salt stress^11^, whereas the rate of growth in *Glycine max* is severely reduced when grown in soils containing up to 0.3% of salt content^12^. Hence, the genetic material of *G. soja* was considered a best choice to elucidate the salt-drought mediated stress signaling networks.

In this work, a novel U-box domain protein gene, *GsPUB8*, was isolated and cloned from wild soybean (*G. soja* 07256). GsPUB8 protein expression was induced by the treatments of high concentrations of NaCl, NaHCO_3_, ABA, and PEG, as indicated by real-time PCR assays. Furthermore, the tissue-specific analysis was performed to study the expression of ubiquitous proteomes and bioinformatics tools were employed to analyze GsPUB8 protein/gene.

## Material and methods

### Plant materials and growth conditions

The explant used in the current study was wild soyabean seeds (*G. soja* 07256) which were collected from the Jilin-Academy of Agricultural Sciences (Chang-Chun, China). It has been confirmed that the experimental samples of plants, including the collection of plant material, complied with relevant institutional, national, and international guidelines and legislation with appropriate permissions from District authorities of Jilin-Academy of Agricultural Sciences (Chang-Chun, China) for the collection of plant specimens. Seeds of *G soja* were surface sterilized for 10–15 min with 98% sulfuric acid and washed 5–6 times with sterilized water to thoroughly remove the acid from seeds before sowing and placed under dark for germination. After two days, seeds were shifted to a growth-chamber with an application of ¼-strength Hoagland's solution. After 21 days, soybean seedlings were shifted into another fresh ¼-strength Hoagland's solution supplemented with 200 mM sodium chloride (NaCl), 50 mM sodium bi-carbonate (NaHCO_3)_, 30% (w/v) PEG6000 and 100 µL abscisic acid (ABA) for stress treatments, while a group of plants without stress treatments was used as a control. Samples of roots and leaves were collected at defined time points. Plant samples were immediately frozen with liquid nitrogen and stored at − 80 °C. For tissue/organ-specific analysis, the sterilized seeds were placed in Petri dishes and placed in complete darkness for about 2–4 days and then transferred into the soil under optimum growth conditions. Plant material was collected at the adult flowering stage of *G. soja* (approximately 2-month-old plants) and preserved in liquid nitrogen at − 80 °C.

### RNA extraction, cDNA synthesis, and isolation of *GsPUB8* gene

Plant RNA was extracted from seedlings of *G. soja* using the Plant Mini Kit (Qiagen). cDNA was generated with the use of the Reverse-Transcriptase kit by following the manufacturer's instructions. For cloning, the *GsPUB8* gene from *G. soja*, the full-length CDS sequence of *GsPUB8* was amplified via PCR method by using gene-specific primers (Table 1). The product of PCR was inserted into pGEM-T Easy Vector, Promega and transformed into DH5α strain of *E. coli* for sequencing of desired gene fragments.

**Table 1:** List of primers designed for cloning of GsPUB8 gene and qRT-PCR assays.

Name of gene	Primers
*GAPDH*	(F 5′-GACTGGTATGGCATTCCGTGT-3′)
	(R 5′-GCCCTCTGATTCCTCCTTGA-3′)
*GsPUB8-1*	(F 5′-AAACCTCGTAAAGGCTTCTCACTAAC-3′)
	(R 5′-AAACTGACCCTTCTCCATTCCATA-3′)
*GsPUB8-2*	(F 5′-AAACCTCGTAAAGGCTTCTCACTAA-3′)
	(R 5′-TTTTCAGACAGGAAGGAAGCATAA-3′)

### Bioinformatics analysis of *GsPUB8* gene

A number of bioinformatics tools and websites were used for sequence comparison and in-silico analysis of GsPUB8 protein/gene such as NCBI Gene Bank database^1^, phytozome^2^, MEGA 4.0 software^3,13^, Clustal Omega^4^, ProtParam tool^5^, SOPMA tool^6^, Softberry^7^, Psort^8^, Gene Structure Display Server^9^ (GSDS; http://gsds.gao-lab.org/), ProtScale^10,14^, DeepLOC-1-0^11^, LocTree3^12^ and SignalP-3 was used to predict signal peptides in the given protein^13^ (Supplementary materials Table 1).

### Quantitative real time PCR

Total RNA was extracted and transcribed into cDNA as discussed above in the methods section. Then, quantitative real time PCR experiments were conducted on an ABI-7500 sequence detection system (Applied Biosystems, Carlsbad, CA, USA). Relative-intensities were normalized and calculated by the methods reported by Willems and his colleagues^15^. Three technical repeats and three fully independent biological replicates were performed to reduce the possible error for statistical analysis.

### Homology modeling and structure prediction

For the prediction of protein model, *GsPUB8* gene sequence was retrieved via Blast search (https://www.uniprot.org/blast). The result showed 54% and 32% sequence identity to U-box domain containing protein 8 and 12 (UniProt ID: O81902 and Q5VRH9) from *Arabidopsis thaliana* and *Oryza sativa* subsp. *japonica,* respectively. The FASTA sequence of U-box domain containing protein 8 of *Arabidopsis thaliana* was consequently used for the 3D modeling of U-box domain containing protein. Therefore, the primary sequence of U-box domain containing protein was subjected to model building via the Swiss Model server^16,17^. The 3D structure of active GID E3-ubiquitin ligase complex minus Gid2 and delta Gid9 RING-domain (PDB: 6SWY) was identified as the most suitable template with 14.50% sequence identity. The SWISS model quality was analyzed via drawing of Ramachandran plot with Procheck software (http://services.mbi.ucla.edu/PROCHECK/)^18^. The images of the predicted model were prepared applying PyMOL^19^.

## Results

### Isolation and sequence analysis of *GsPUB8* from *G. soja*

**Figure 1:**
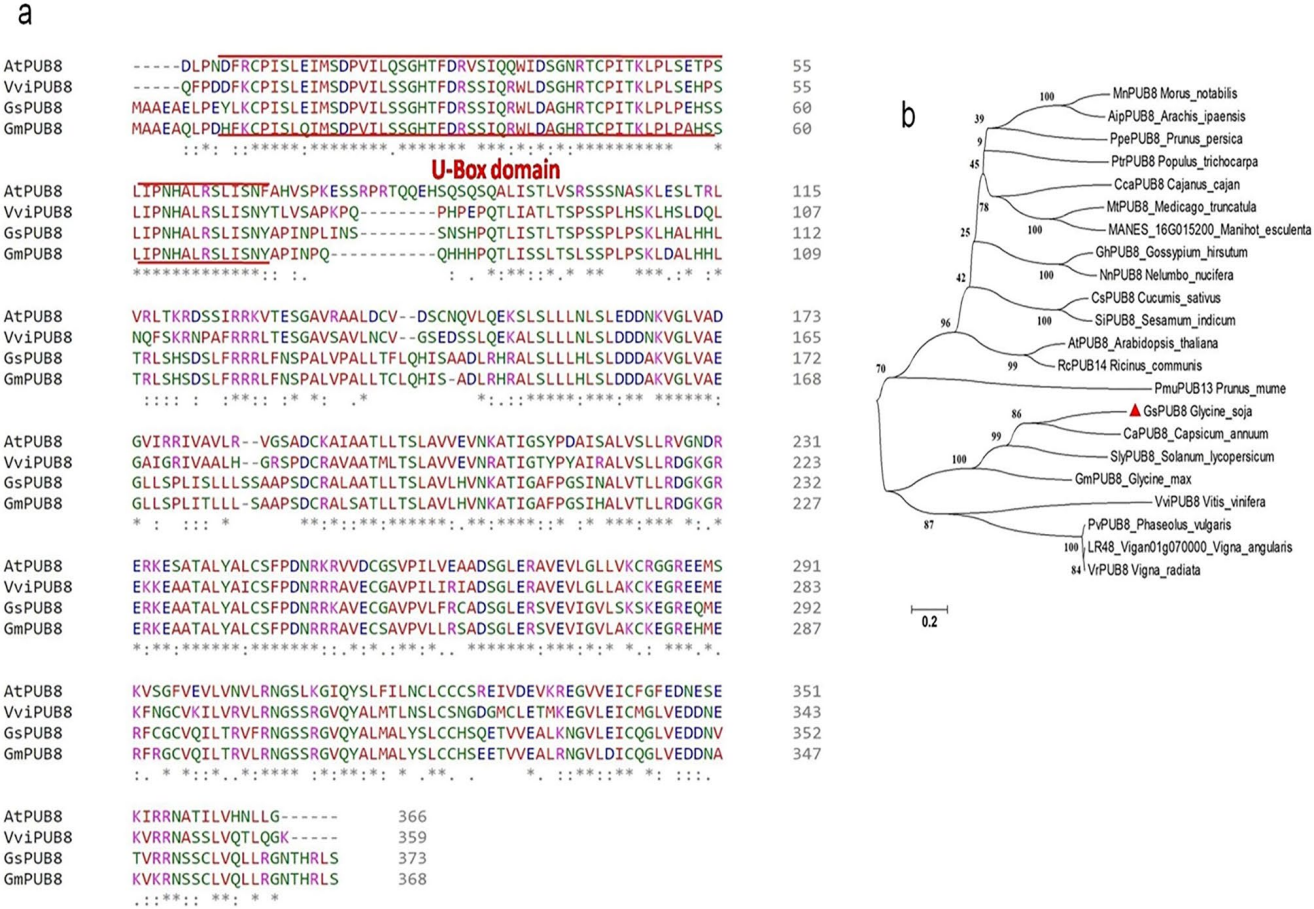
The sequence analysis of GsPUB8 protein. (**a**) The multiple sequence-alignment analysis of GsPUB8 amino acid sequences. (**b**) A phylogenetic tree highlighting close relationships among PUB8 protein from *G. soja* and other PUB8 proteins of monocots and dicots.

Previously, many stress-related genes, including *GsPUB8* (*Glyma13g32290*), induced by alkali stress conditions were obtained through transcriptome sequencing of wild soybean (*G. soja* 07256) roots^20^; thus, *GsPUB8* was further characterized in terms of In-silico and expression levels under different abiotic stress conditions. For this purpose, specific primers were used for the amplification of full-length *GsPUB8* cDNA through a homology-based cloning method, presented by MacNeil and Weinberg^21^. The results of amino acid sequencing revealed that *GsPUB8* has an open reading frame (ORF) of 1122 bp, encoding a protein consisting of 373 amino acid residues (average molecular weight, 40,562 Da; Supplementary materials Fig. 1). Multiple alignment sequence-analysis were executed to elucidate the accurate relationship between the GsPUB8 protein and its associated PUB8 proteins in different plants, including *G. max*, *Vitis vinifera* and the model plant *A. thaliana*. GsPUB8 protein is 65–95% similar to PUB8 proteins of the above mentioned species based on the entire sequence alignments. The predicted conserved domain and multiple sequence alignment analysis revealed that the GsPUB8 protein contains one typical U-box domain (a modified RING finger) at the N-terminus (10–74 amino acid interval) (Fig. 1a). To verify the evolutionary associations within plant U-box domain proteins, a phylogenetic study was executed by using MEGA-4.0 software^13^ based on amino acid sequences of GsPUB8 and homologs from diverse monocot-dicot plants (Fig. 1b). These results indicate that GsPUB8 clusters with other dicot plants in the same branch that contains homologs of *Capsicum annum*, *Solanum lycopersicum*, *Glycine max*, *Vitis vinifera*, *Phaseolus vulgaris*, *Vigna angularis* and *Vigna radiata*. The distribution patterns of PUB8 proteins in angiosperms could further highlight its evolutionary nature as well as diverse functions in higher plants.

### *GsPUB8* gene structure analysis

**Figure 2:**
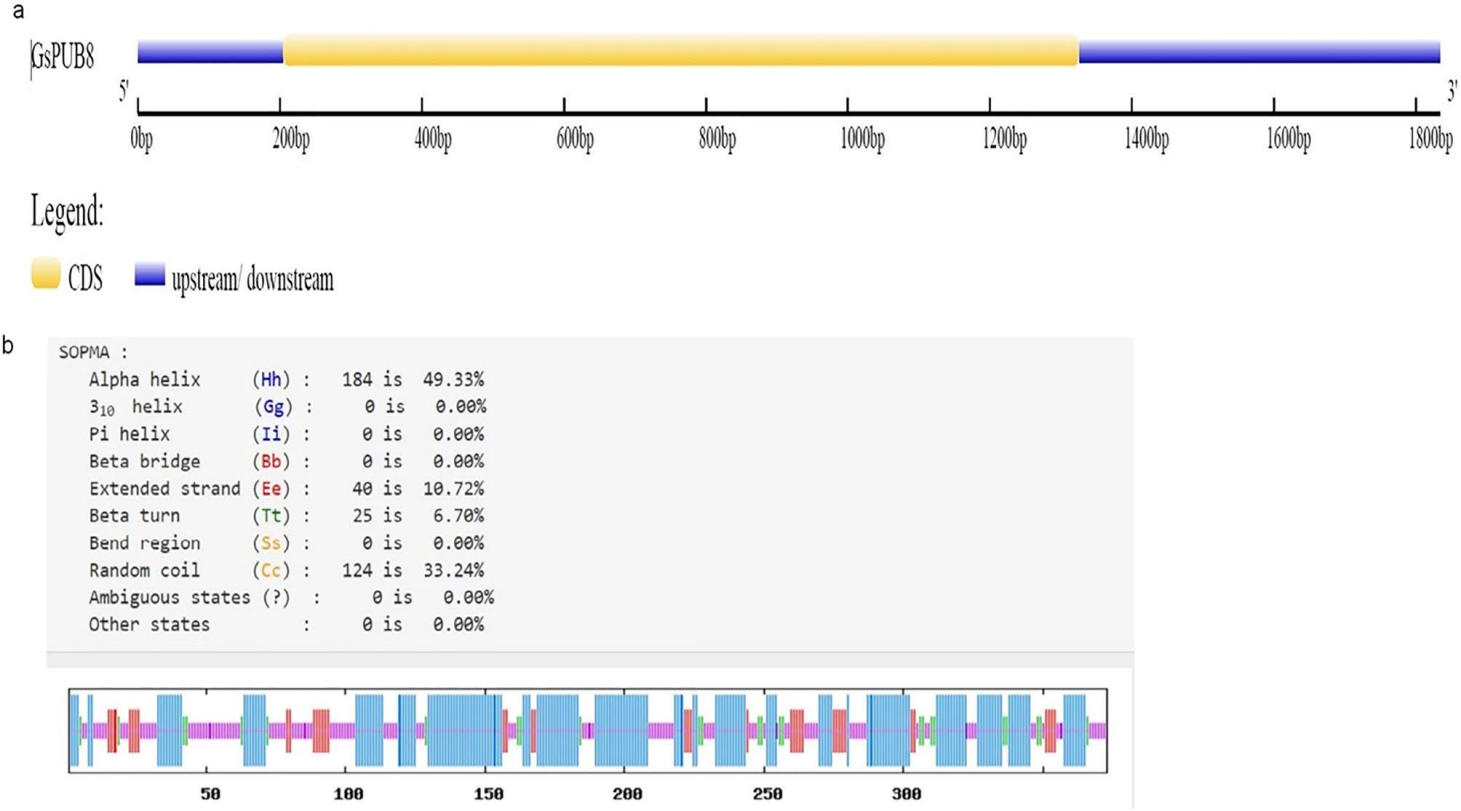
The gene structure analysis of GsPUB8 protein. (**a**) Identification of exon/intron organization. (**b**) The diagrammatic representation of GsPUB8 secondary structure.

In order to gain further insights into the structural diversity of *G. soja PUB8* genes, an online software^9^ was used to display the structure of an exon/intron structure (Fig. 2a). The results revealed that the *GsPUB8* gene has no intron in its coding region and is composed of only one exon. Other homologous genes of *GsPUB8*, such as *GmPUB8*, *VviPUB8* and *MtrPUB8*, also showed a similar pattern of gene structure with no intron and one exon (data not shown). Furthermore, the secondary structure of GsPUB8 protein by using SOPMA tool revealed that the GsPUB8 protein comprised of 184 α-helix (49.33%), 40 extended strands (10.72%), 25 Beta turns (6.70%) and 124 random coils (33.24%) (Fig. 2b). Our results show the absence of introns in the ORF of the GsPUB8 gene, which indicates the pure nature of this gene.

### Physico-chemical and hydrophobicity properties

**Figure 3:**
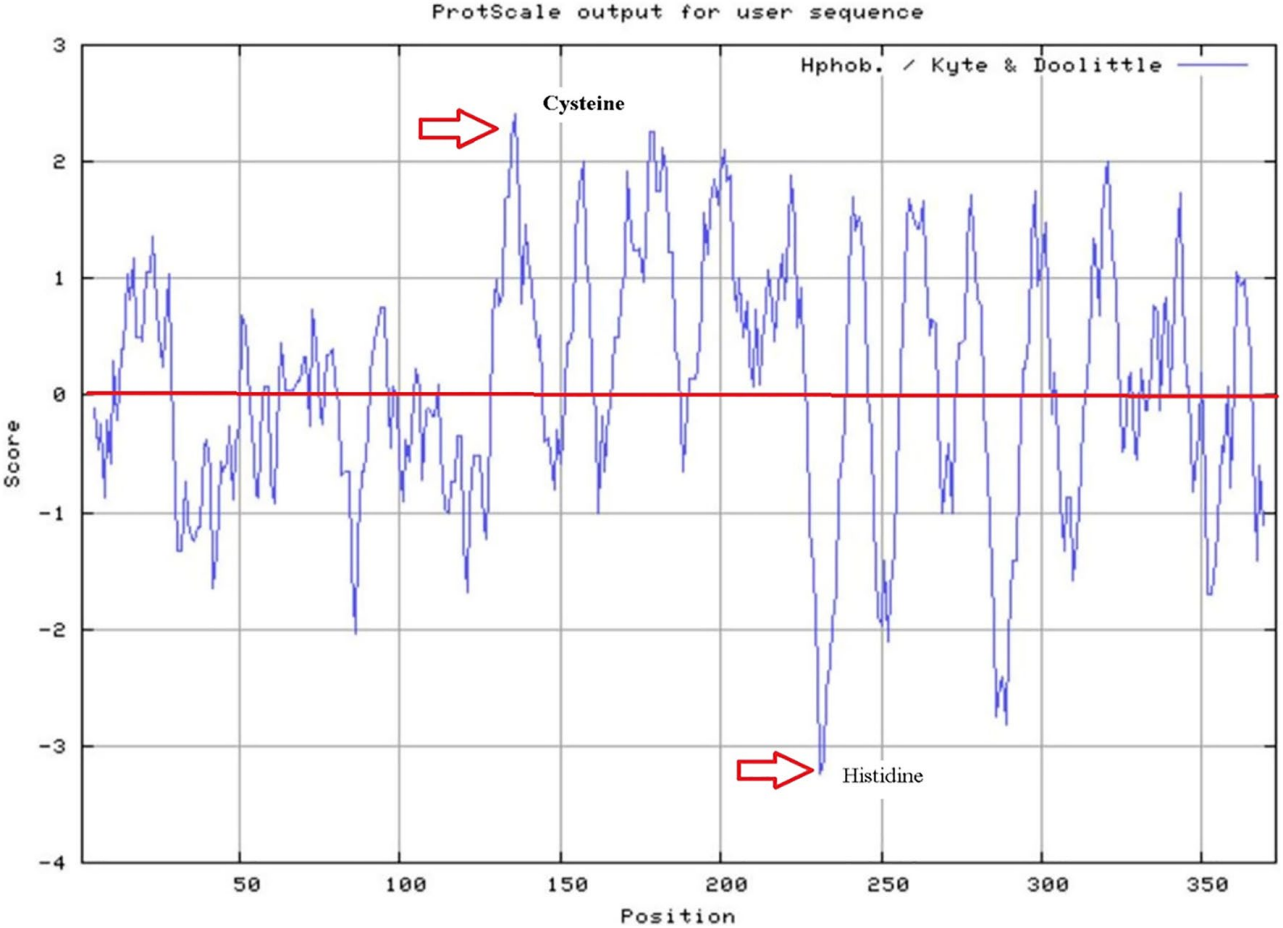
The representation of hydrophobic–hydrophilic patterns in GsPUB8 protein by use of Protscale software. The hydrophobic and hydrophilic domains are considered to exist above and below the zero line respectively.

The primary characterization analysis of the GsPUB8 protein was done by using the ProtParam tool (Supplementary materials Table 2). To confirm the hydrophobicity patterns of the GsPUB8 protein, ProtScale software was used. As shown in Fig. 3, the lowest recorded point was − 3.233 at position 231 (Histidine) in the GsPUB8 polypeptide chain, while the highest score recorded was 2.41 at position 136 (Cysteine). These are identified as the strongest and weakest points of hydrophobicity.

### Predicted sub-cellular localization of GsPUB8 protein

**Figure 4:**
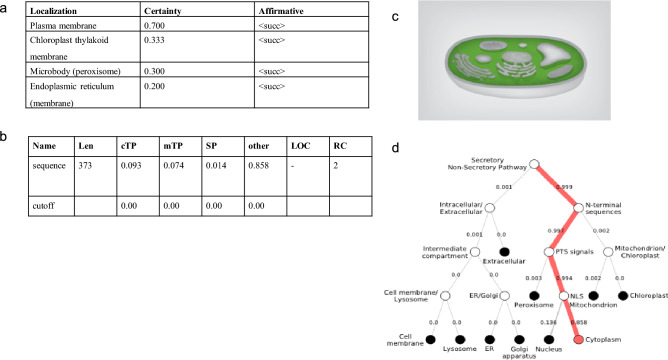
The predicted protein subcellular location results (**a**) by using Psort, (**b**) TargetP; mitochondrial targeting peptide, SP, cTP; chloroplast transit peptide, mTP; secretory pathway, (**c**) LocTree3, (**d**) DeepLoc.1.0.

In our study, the Soft berry and Psort software packages were used to examine the predicted subcellular localization of the GsPUB8 protein and it showed the highest activity in plasma membrane (Fig. 4a). While, Fig. 4b showed that the GsPUB8 protein might not be actively involved in secretory, photosynthetic or metabolic pathways, as this protein lacks significant values of signaling peptides in the above-mentioned pathways. Furthermore, DeepLoc-1.0 and LocTree 3 software’s were used to investigate the GsPUB8 protein intracellular localization. Results showed that GsPUB8 protein was mainly located in the cytoplasm and in non-secretory pathway (Fig. 4c,d).

### Expression patterns analysis of *GsPUB8*

**Figure 5:**
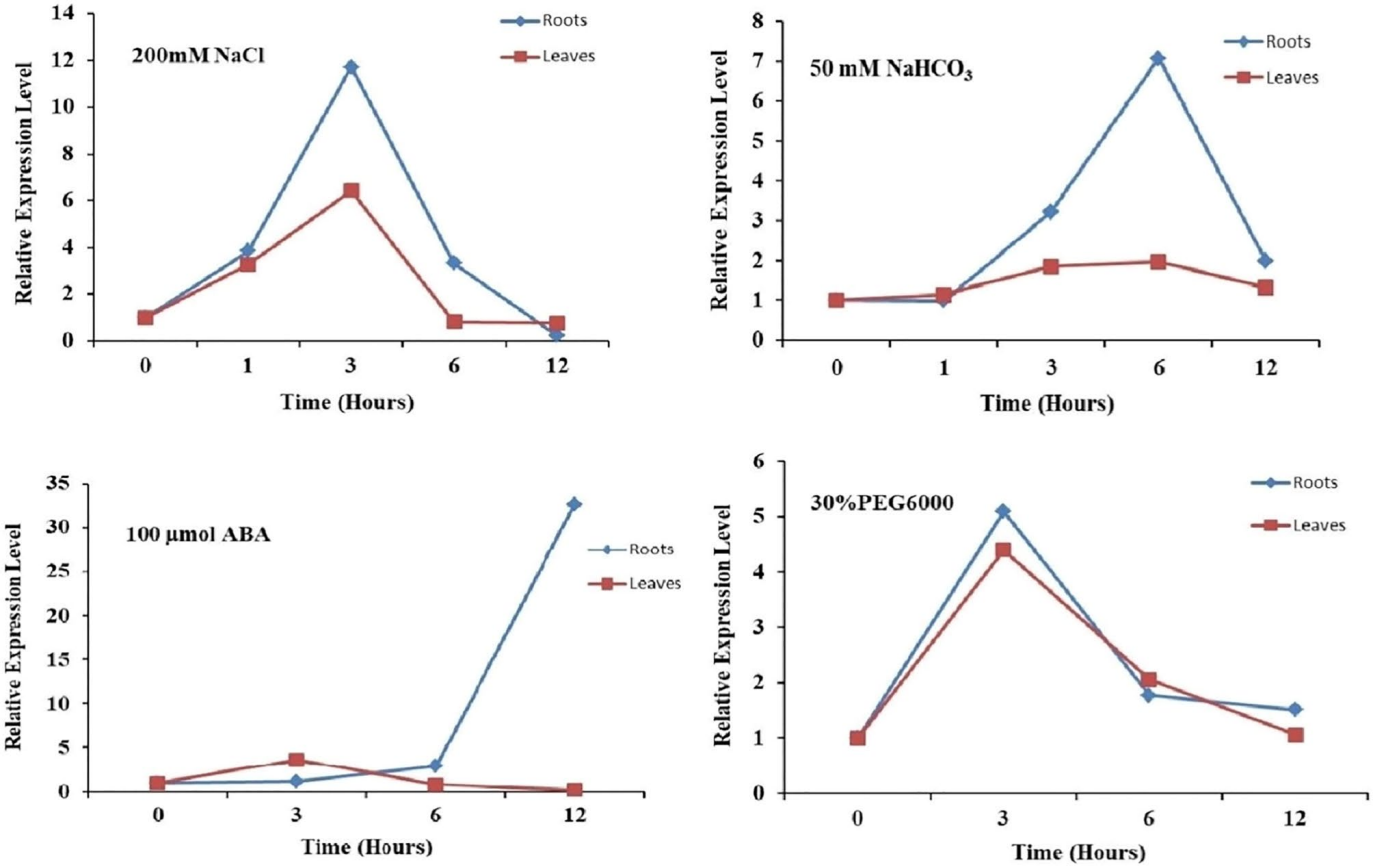
Expression patterns of *GsPUB8* in *G. soja* under the application of salt, alkali, ABA and osmotic stress. *GsPUB8* expression was assessed by qRT-PCR via 2^−ΔΔCT^ method. Here, the error bars signify the ± SE (n = 3).

An approach of Real time q-PCR was performed to clarify the more comprehensive results concerning the stress response expressions of the *GsPUB8*. The expression levels of *GsPUB8* increased dramatically in response to 100 µM ABA and attained a maximum peak at 12 h in the roots (32-fold) and leaves (3.5-fold) in a similar manner (Fig. 5). However, the *GsPUB8* expression level was analogously low under the osmotic stress treatment with polyethylene glycol for both roots and leaves (5.2-fold and 5-fold) respectively. Furthermore, the expression of *GsPUB8* significantly increased with 200 mM salt treatment at 1 and 3 h in both roots (12-fold) and leaves (6.2-fold) respectively while, under NaHCO_3_ treatment at higher expression noted at 6 h in the roots (7-fold) and leaves (1.8-fold).

### Expression analysis of *GsPUB8* in multiple organs of *G. soja*

**Figure 6:**
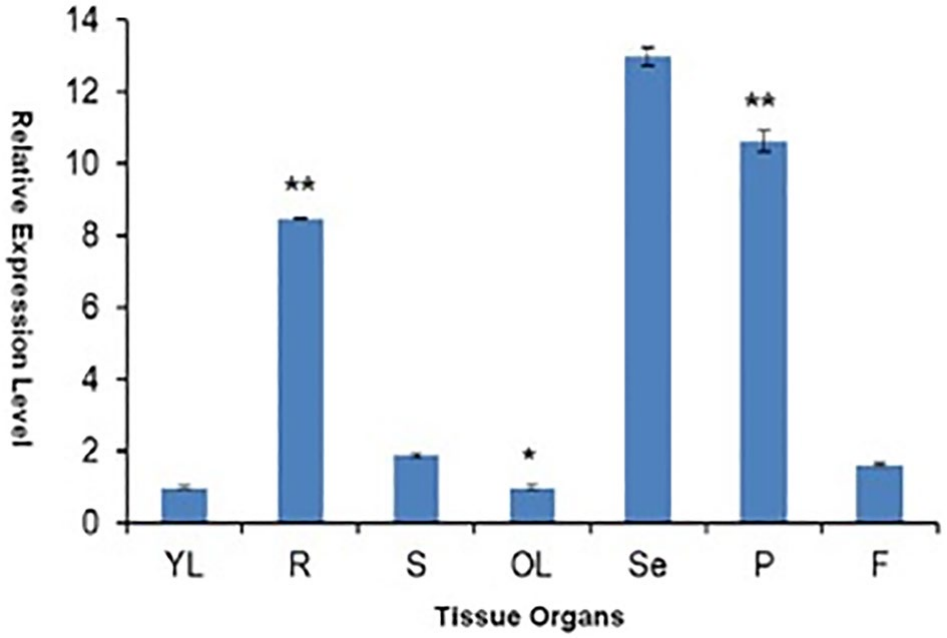
The tissue-specific expression pattern analysis of the GsPUB8 gene in multiple organs of *G. soja*. The plant organs used in this experiment included (YL) young-leaf, (S) stem, (R) root, (OL) old-leaf, (F) flower, pod (P) and (Se) seed. Error bars signify the ± SE (n = 3).

The tissue-specific expression-levels of *GsPUB8* in various tissues from mature *G. soja* plants was investigated through a quantitative RT-PCR assays. *GsPUB8* was expressed in both reproductive and vegetative organs, e.g., roots, leaves, pods, stems, and flowers and the expression of *GsPUB8* was higher in seeds, pods, and roots then leaves, stems, and flowers (Fig. 6). *GsPUB8* expression zones were consistent in older leaves in contrast to younger leaves. However, more studies are required to understand the functional and regulatory roles of the *GsPUB8* gene in wild soybeans under abiotic stress conditions.

### GsPUB8 protein homology modeling

**Figure 7:**
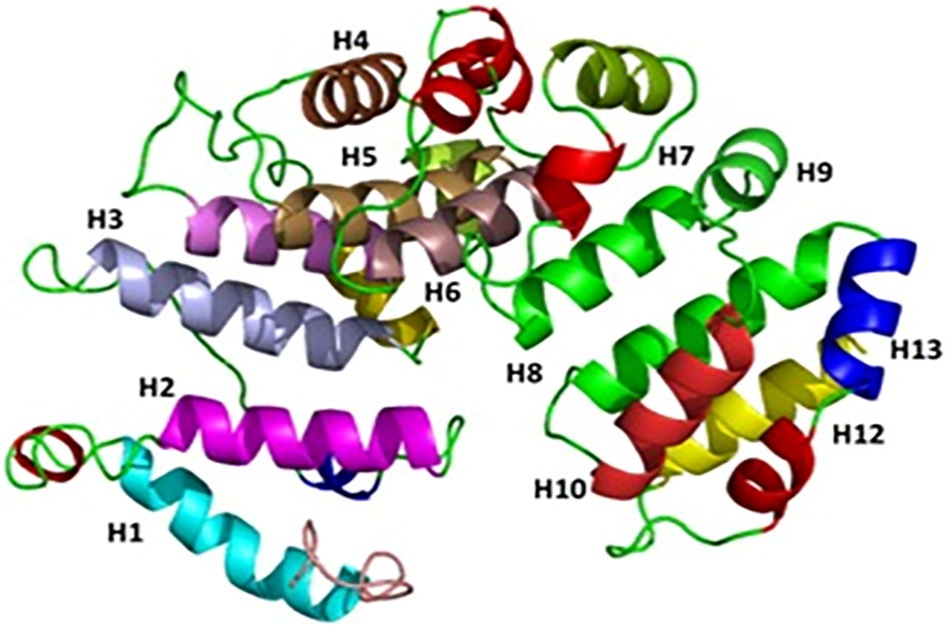
Structural features of GsPUB8 protein from *Glycine soja* indicating U-box domain with franked α-helix.

**Figure 8:**
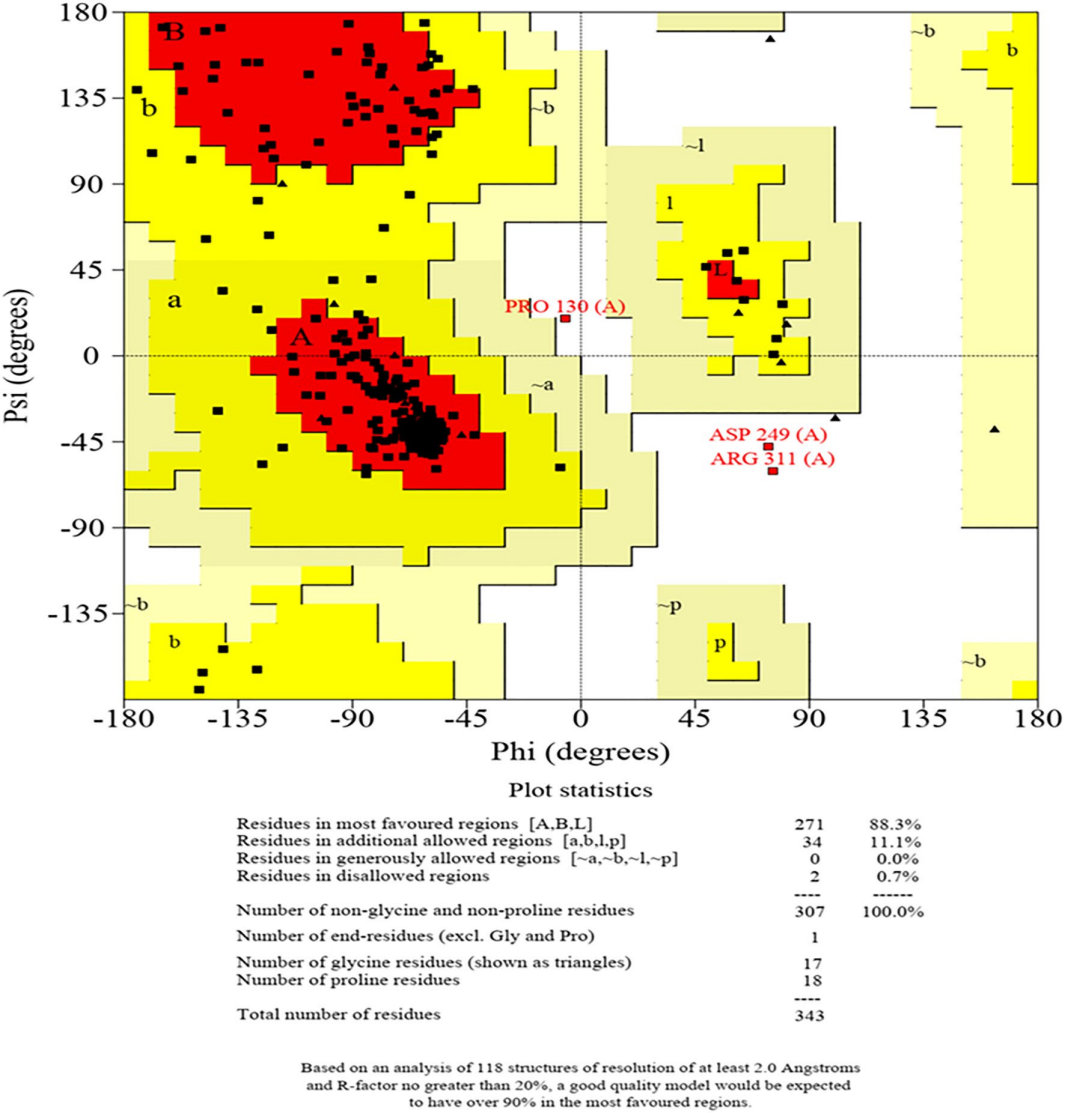
Ramachandran plot of the modeled PUB8 structure indicating two amino acids in the disallowed region as an indication of good quality model.

The *Gs*PUB8 3D model consists of a U-box domain which is main characteristics of this protein. GsPUB8 structure contains a central alpha-helix lined by two surface exposed loops which are arranged in a cross-brace formation. GsPUB8 U-box domains comprised of an antiparallel α-helix type arrangement involving the central alpha helix and first surface exposed loop. Antiparallel α-helix is stabilized by highly conserved hydrophobic residues which are responsible for the stability and core packing of the molecule (Fig. 7). GsPUB8 structure model has an elongated C terminal helix like the other U-box domain structures and its structure quality was evaluated by proCheck online tool which generated a Ramachandran plot. Out of 343 residues, Ramachandran plot of *Gs*Pub8 proteins has 88.3% in the high favored-region, 11.1% in allowed-region, 0% in generously allowed and 0.7% in disallowed-region (Fig. 8). Therefore, the GsPUB8 model structure is good enough for further analysis.

## Discussion

Plant U-box domain-containing proteins (PUB) are composed of 70 amino acid residues and contain a U-box motif, which is considered as an interesting and important feature of this small but biologically active group of proteins. This motif was determined to be a modified RING domain, and a large number of U-box proteins have been shown to work as E3 ubiquitin ligases^3^. Nonetheless, there is limited knowledge in the literature about U-box proteins (E3 Ub-ligases) in plants as compared to mammalian and yeast systems. Recent studies show that this protein family is entering a new era of focus due to their involvement in multiple biological and physiological functions^22,23^. In this regard, our study specifically focused on the GsPUB8 protein gene from wild soybean (*G. soja* 07256) to provide basic information about this gene family. In our study, a novel U-box domain gene was isolated and cloned from the roots of *Glycine soja* 07256. This *GsPUB8* gene has an ORF of 1122 bp that encodes 373 amino acid residues, a molecular weight of 40,562 Da, and the characteristic feature of a 70-amino acid residue U-box domain. The classification criteria of plant U-box E3 ligases is unique because it is based on the occurrence of some additional domains/motifs other than the U-box domain: GKL, ARM/HEAT, TPR, UFD2 and WD40^2^. In a recent study, a total of 125 *GmPUB* genes from *Glycine max* were categorized into eight classes (class I to class VIII) based on the presence of additional motifs^24^. As per this criterion, the fifth class of *GmPUB* gene family, composed of seventeen members, emerged with only a single U-box motif at the N-terminal region with no other protein motif/domain/sequence in that class. As shown in Fig. 2a, our multiple sequence alignment analysis revealed that the GsPUB8 protein belongs to class V of PUB/E3 ligases. Our results clearly indicate the presence of a U-box domain motif at N-terminus. In addition, our phylogenetic analysis (Fig. 2b) showed the highest similarity of GsPUB8 amino acid sequences with other dicots. Our GsPUB8 protein clustered with dicot plants such as *Glycine max* (100%), *Capsicum annum* (57%), *Solanum lycopersicum* (57%), *Vitis vinifera* (67%), *Phaseolus vulgaris* (67%), *Vigna angularis* (67%) and *Vigna radiata* (68%) of the Fabaceae, Solanaceae and Vitaceae plant families. It is notable fact that introns fulfill a broad spectrum of cellular functions playing a role in mRNA processing. In early research in eukaryotes, introns were considered as “junk” elements, but with the passage of time, introns gained attention based on their functions in different eukaryotic lineages. Increasing numbers of introns per gene is a sign of developmental complexity of the genome in many eukaryotes, and higher plants mostly contain five to six introns per gene^25^. To clearly understand the structure of the *GsPUB8* gene, online software (Gene Structure Display Server) was used (Fig. 3). Results of current studies showed the absence of introns in the ORF of the *GsPUB8* gene, which indicate the simple nature of this gene. Previously, it was reported that the subcellular localization of the PUB family proteins was divergent/diverse. A handful of studies have established that the existence of multiple motifs/sequences can directly affect protein localization of PUB proteins such as those with ARM domains. For instance, AtPUB9, StPHOR1, AtPUB13 and BnARC1 mainly reside in the nucleus; BnARC1 is found on the ER (proteasome structures); StPHOR1, AtPUB22, BnARC1, AtPUB23 and AtPUB13 localize in the cytoplasm; and AtSAUL1 resides localize on plasma membranes^26–29^. Meanwhile, PUB proteins with only U-box domains, such as GmPUB8, reside in the cytoplasm and in Golgi compartments^24^. Like the GmPUB8 protein, our results predict the subcellular location of the GsPUB8 protein in the plasma membrane, while target PI results also reveal the presence of GsPUB8 signaling peptides in pathways other than chloroplast, mitochondria, and secretory pathways, which proved the uniqueness of this protein compared to its homologs. Several PUB proteins such as AtPUB22, 23, 18, 19, CaPUB1, and GmPUB8 are reported to have important roles in abiotic stress responses, and all above mentioned proteins also work as E3 ubiquitin-ligases^24,30–32^. In accordance, our real-time qPCR assay data showed that *GsPUB8* transcripts exhibit significant changes in response to salt, alkali, ABA, and osmotic stress treatments, which suggest a major contribution of GsPUB8 during regulation of plant abiotic stress responses (Fig. 5). Recent reports on other PUB family genes such as *GmPUB6*, *TaPUB1*, *TaPUB15* and *CsPUB88* also showed higher expression levels in response to salinity, drought, cold, pathogens and different phytohormones treatments in soybean, wheat and tea plants respectively^33–36^.

Similarly, the tissue-specific expression analysis of the *GsPUB8* gene reveals that a peculiar expression of GsPUB8 exists in *G. soja*. Higher levels of GsPUB8 transcripts were observed mainly in the seeds, pods, and roots (Fig. 6). In contrast, these transcripts were hardly detected in stems or flowers or in young and old leaves. These findings suggest that the *GsPUB8* gene could play an essential role in soybean growth and development but not necessarily in reproduction and these results are in accordance to the findings of Wang et al.^24^, which showed the highest expression levels of GmPUB8 in roots compared to other tissues. The structure quality of GsPUB8 protein was estimated by using an online tool proCheck and a Ramachandran plot was generated which showed that most of the GsPUB8 proteins are in a favored region (88.3%) as well as GsPUB8 structure model has central *a*-helix and an elongated C-terminal helix like the other U-box domain structures^30^. As previous studies reported, that the L1–α1–L2 motif of RING and U-box domains are essential elements during E2 interactions^27,37–39^.

The purpose of this study was to clarify the stress tolerance functions of genes in wild Soybean. Based on these results, it is expected that *GsPUB8* gene could be used as a target for marker development in soybean. However, the functional and regulatory mechanisms of this gene needs to be further explored after its transformation in to other model spp like *Arabidopsis* and Tobacco.

## Conclusion

*GsPUB8*, a novel member of the plant U-box domain family in wild soybean, was isolated by a homology-based cloning method. An In-silico characterization analysis was performed to get insights into the structure of the *GsPUB8* gene. *Gs*PUB8 protein 3D model structure was good enough for its further characterization. Besides, qRT-PCR studies revealed the expression of the *GsPUB8* gene in both reproductive and vegetative organs of soybean, and soybean roots exhibited induced expression levels under salinity and drought stress as compared to leaves. This study could help in providing valuable information about the possible functional roles of *GsPUB8* in legumes under abiotic stresses. The next research plan after these findings is to further explore the mechanisms of this gene in wild soybean. For this purpose, constitutive overexpression studies of this gene in model plants might be a good way for its functional characterization specifically under stress conditions.

## Supplementary Information

Supplementary Information 1.

Supplementary Information 2.

## Data Availability

The datasets generated and/or analysed during the current study are available in the manuscript.

## Abbreviations

ABA: Abscisic acid
PUB8: Plant U-box domain protein 8
RING: Really interesting new gene
PEG: Polyethylene glycol
