# Supplementary material for: Cloning and in silico characterization of an abiotic stress-inducible U-box domain-containing protein gene GsPUB8 from Glycine soja
Source: Sci Rep. 2022 Oct 13;12:17146. doi: 10.1038/s41598-022-21583-9 (PMC9561723; doi:10.1038/s41598-022-21583-9)

## Slide 1
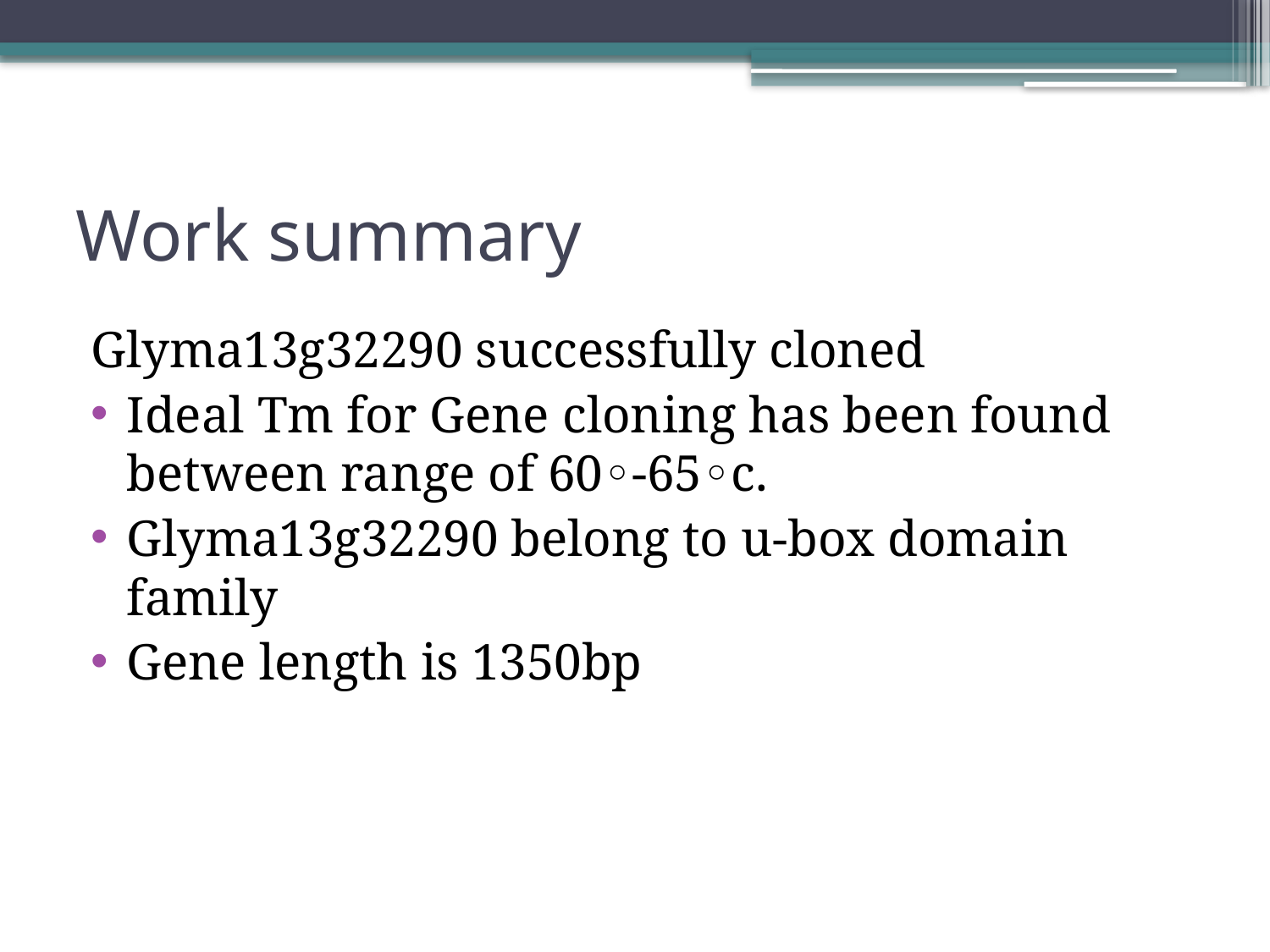

# Work summary
Glyma13g32290 successfully cloned
Ideal Tm for Gene cloning has been found between range of 60◦-65◦c.
Glyma13g32290 belong to u-box domain family
Gene length is 1350bp

## Slide 2
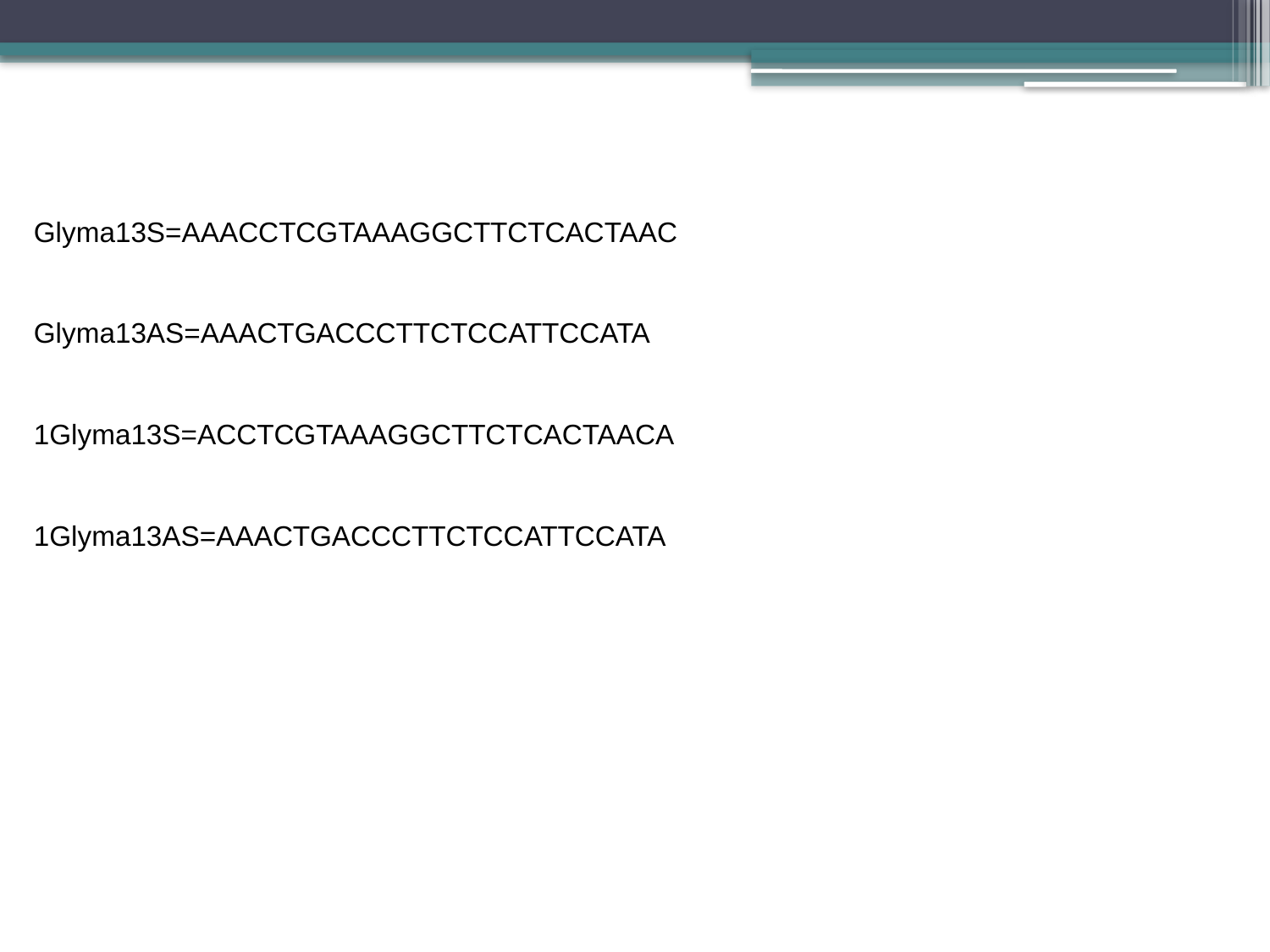

Glyma13S=AAACCTCGTAAAGGCTTCTCACTAAC
Glyma13AS=AAACTGACCCTTCTCCATTCCATA
1Glyma13S=ACCTCGTAAAGGCTTCTCACTAACA
1Glyma13AS=AAACTGACCCTTCTCCATTCCATA

## Slide 3
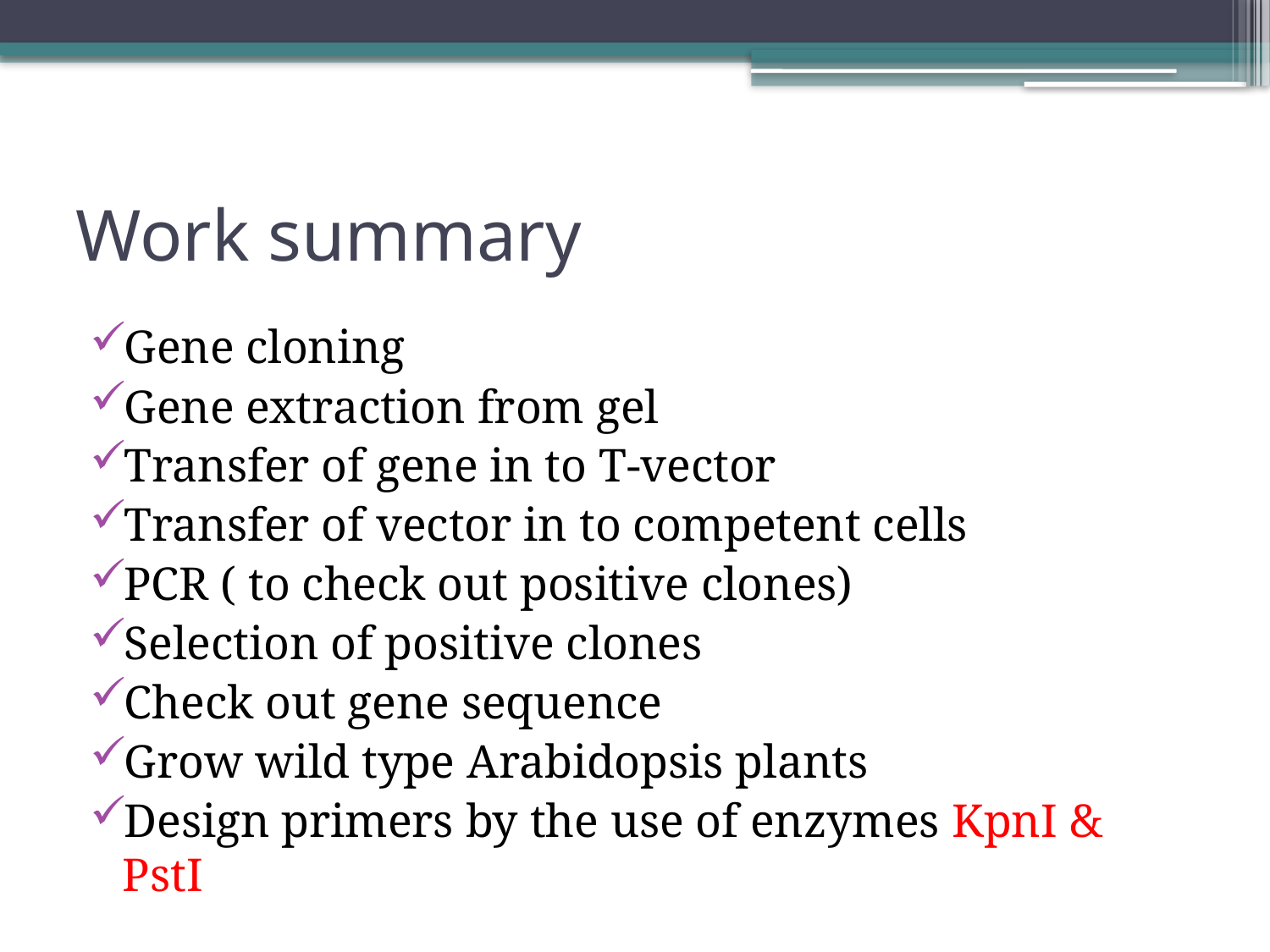

# Work summary
Gene cloning
Gene extraction from gel
Transfer of gene in to T-vector
Transfer of vector in to competent cells
PCR ( to check out positive clones)
Selection of positive clones
Check out gene sequence
Grow wild type Arabidopsis plants
Design primers by the use of enzymes KpnI & PstI

## Slide 4
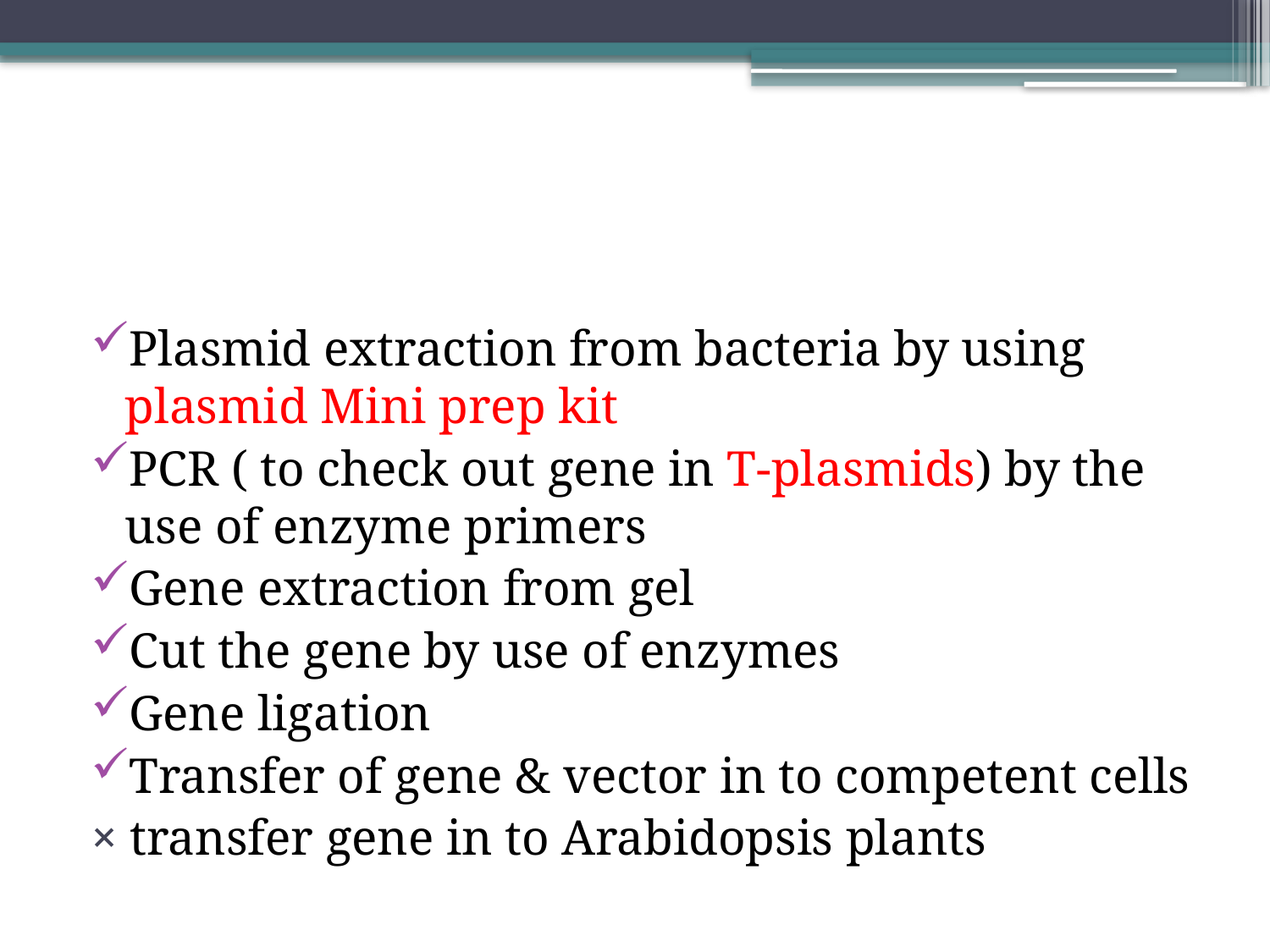

#
Plasmid extraction from bacteria by using plasmid Mini prep kit
PCR ( to check out gene in T-plasmids) by the use of enzyme primers
Gene extraction from gel
Cut the gene by use of enzymes
Gene ligation
Transfer of gene & vector in to competent cells
× transfer gene in to Arabidopsis plants

## Slide 5
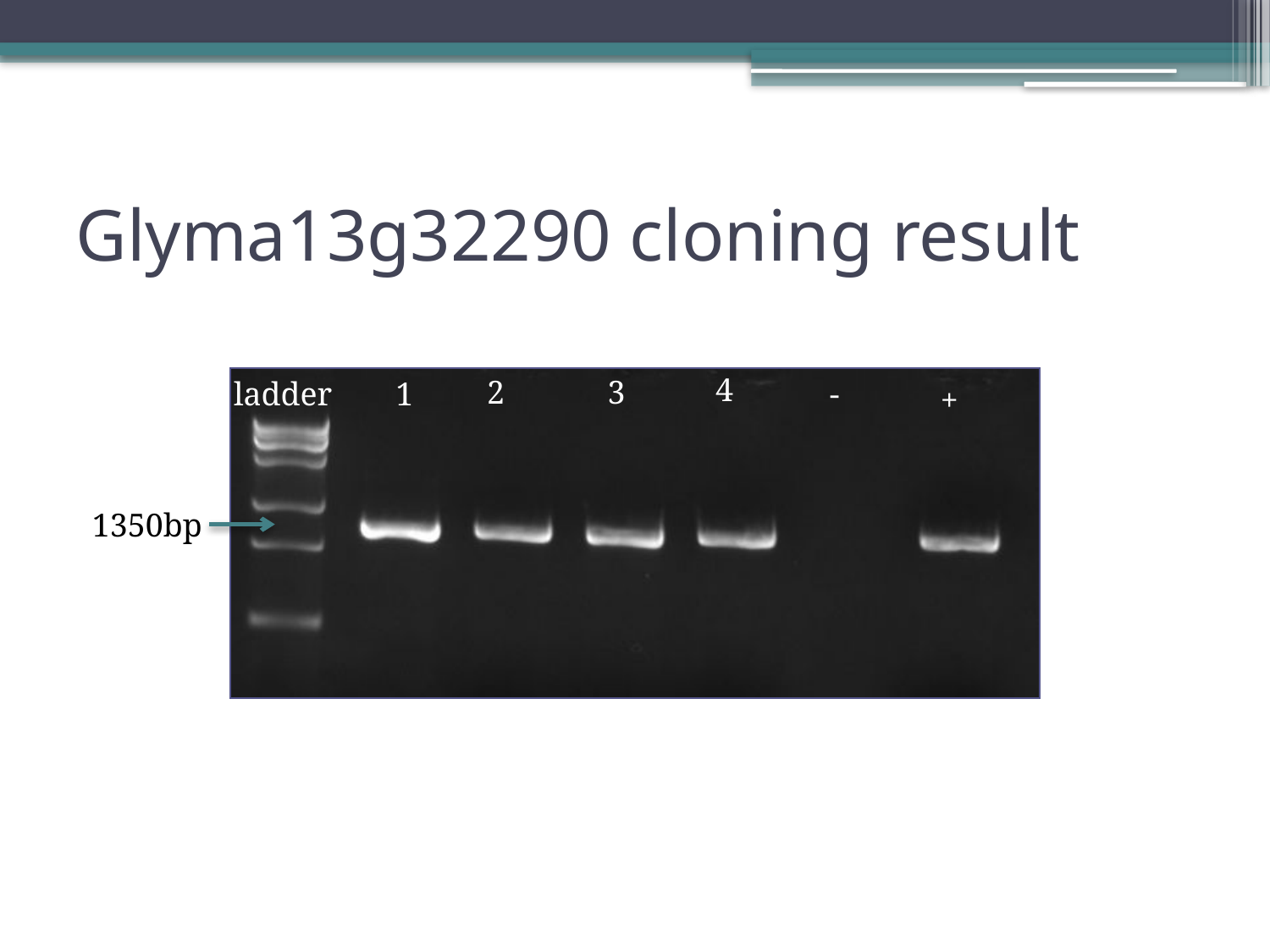

# Glyma13g32290 cloning result
4
2
3
ladder
1
-
+
1350bp

## Slide 6
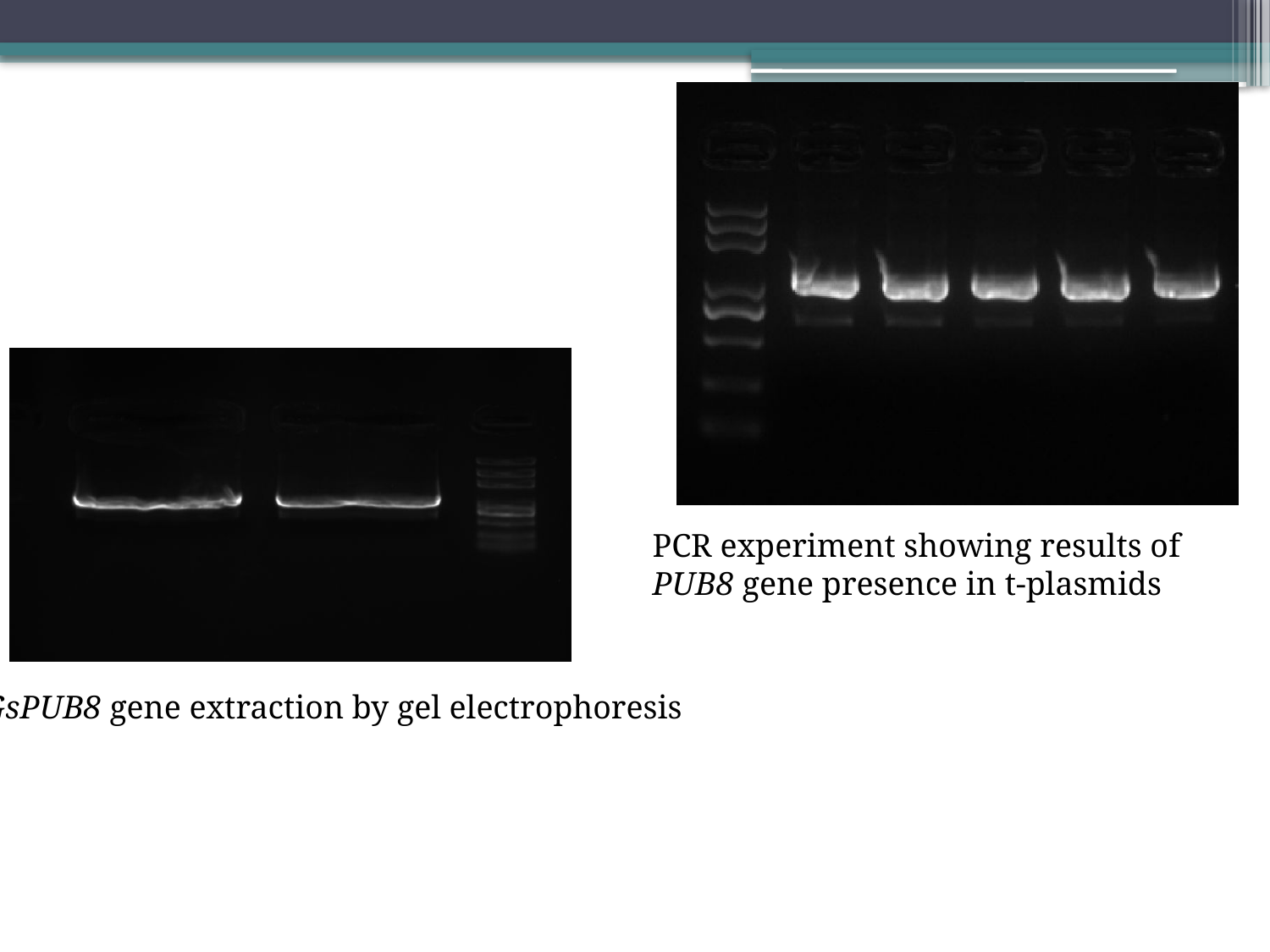

PCR experiment showing results of PUB8 gene presence in t-plasmids
GsPUB8 gene extraction by gel electrophoresis

## Slide 7
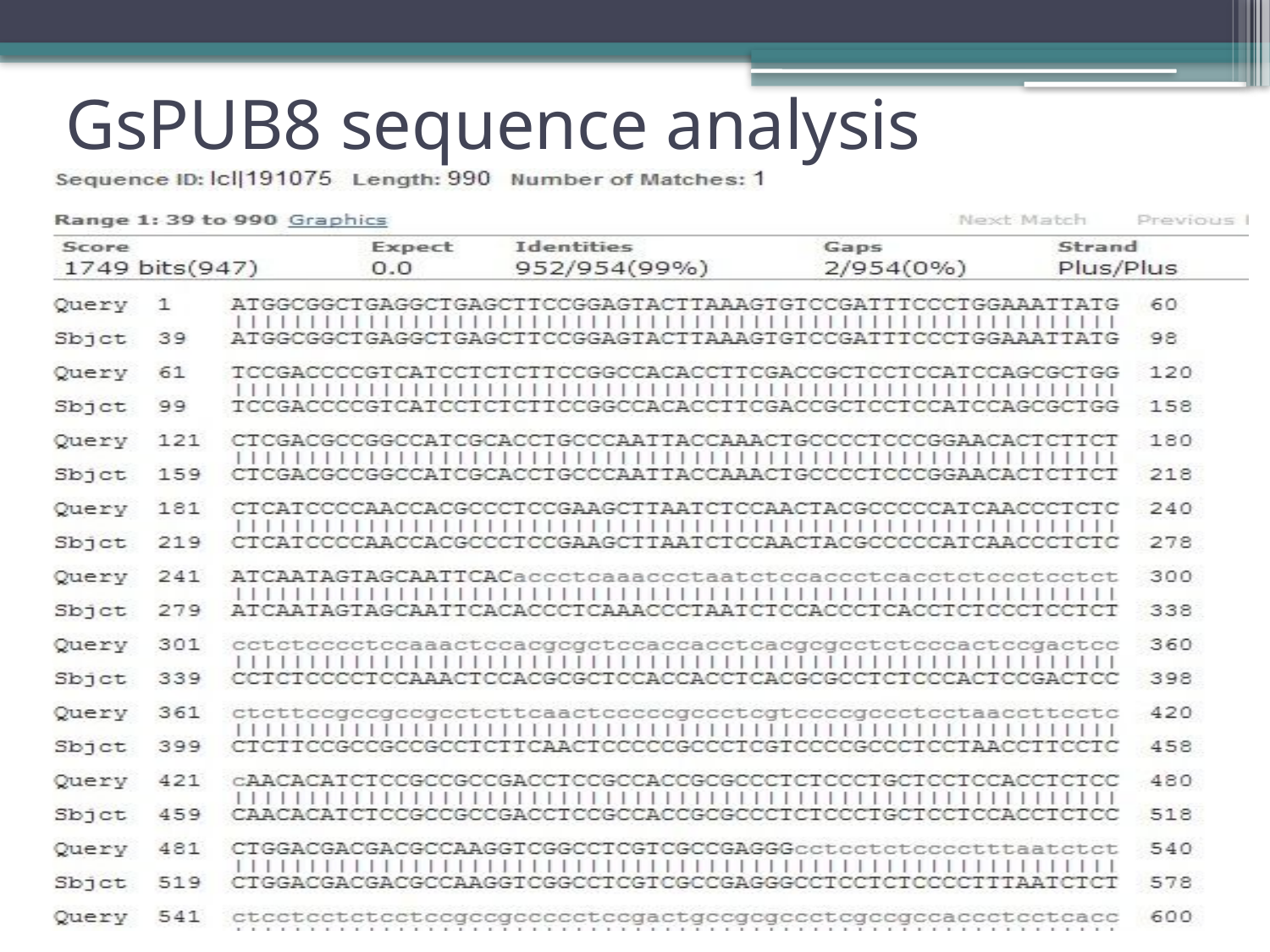

# GsPUB8 sequence analysis

## Slide 8
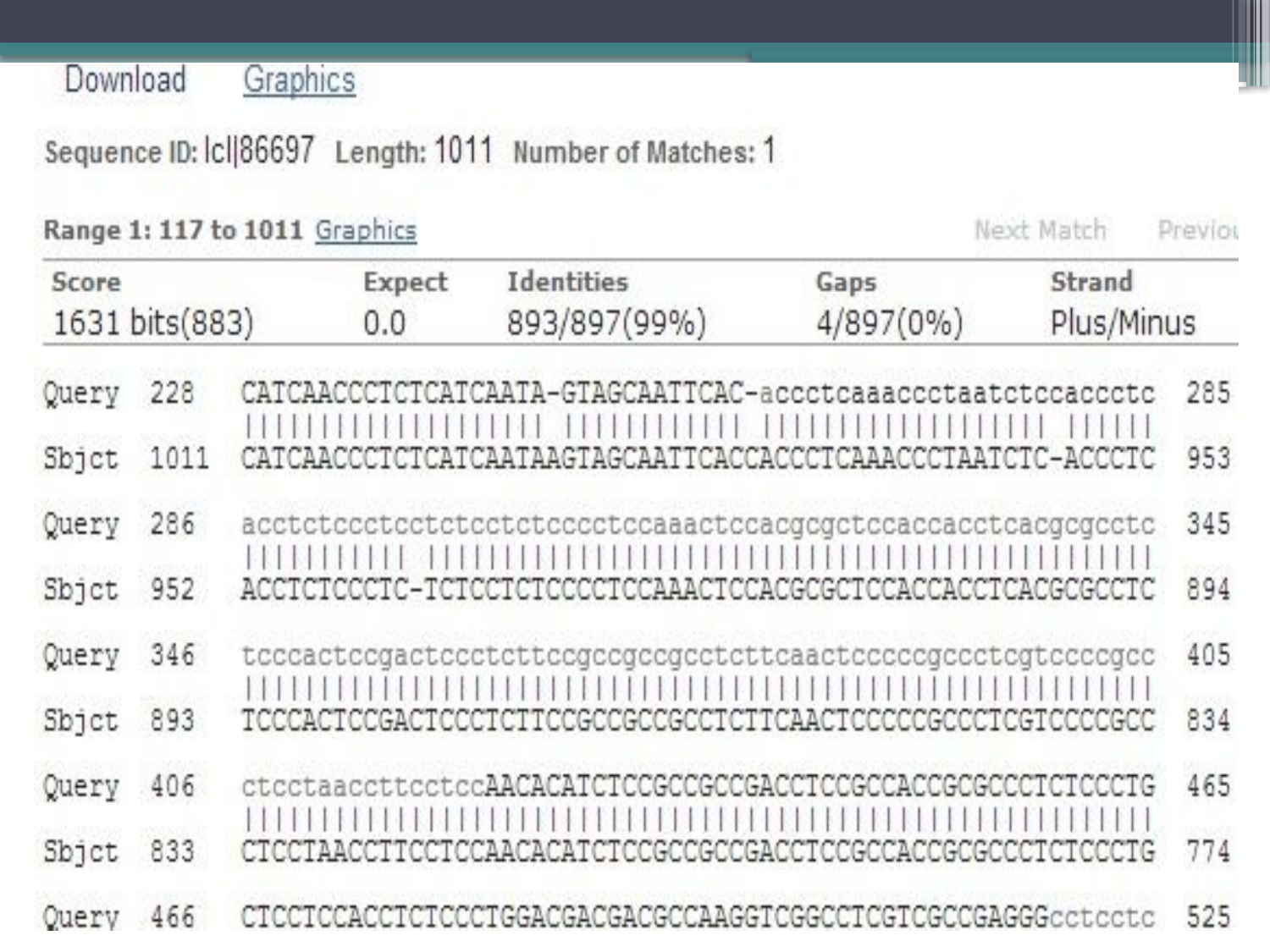

Supplement: Supplementary file 1 — Supplementary Information 1. [file 41598_2022_21583_MOESM1_ESM.pptx]
