# Supplementary material for: Cloning and in silico characterization of an abiotic stress-inducible U-box domain-containing protein gene GsPUB8 from Glycine soja
Source: Sci Rep. 2022 Oct 13;12:17146. doi: 10.1038/s41598-022-21583-9 (PMC9561723; doi:10.1038/s41598-022-21583-9)
Supplement: Supplementary file 2 — Supplementary Information 2. [file 41598_2022_21583_MOESM2_ESM.docx]

**Supplementary materials tables and figures**

Table **1**. Detail of various bioinformatics websites/tools used to analyse GsPUB8 protein structure and function

| Sr No | Websites |
| --- | --- |
| 1  2  3  4  5  6  7  8  9  10  11  12  13 | 1.http://blast.ncbi.nlm.nih.gov/Blast.cgi  2.http://www. phytozome.net/soybean  3. https://www.megasoftware.net/mega4/  4. http://www.ebi.ac.uk/Tools/msa/clustalo/  5. http://web.expasy.org/protparam/  6.https://npsaprabi.ibcp.fr/  7.http://www.softberry.com  8. http://psort.hgc.jp  9. http://gsds.cbi.pku.edu.cn/  10.http://web.expasy.org/protscale/  11. http://www.cbs.dtu.dk/services/DeepLoc/  12. https://rostlab.org/services/loctree3/  13. http://www.cbs.dtu.dk/services/SignalP |

S Table 2: List of some Physio-chemical properties of GsPUB8 protein done by using the ProtParam tool.

| Sr No | Physico-Chemical Properties |  |
| --- | --- | --- |
| 1 | Molecular formula | C_1782_H_2921_N_519_O_527_S_16_ |
| 2 | Isoelectric point (pI) | 8.73 |
| 3 | Molecular weight | 40561.92 Da |
| 4 | Instability index | 54.86 |
| 5 | *In vitro* half-life | 30 hours |
| 6 | Aliphatic index | 109.33 |
| 7 | Hydropathicity index | 0.087 |


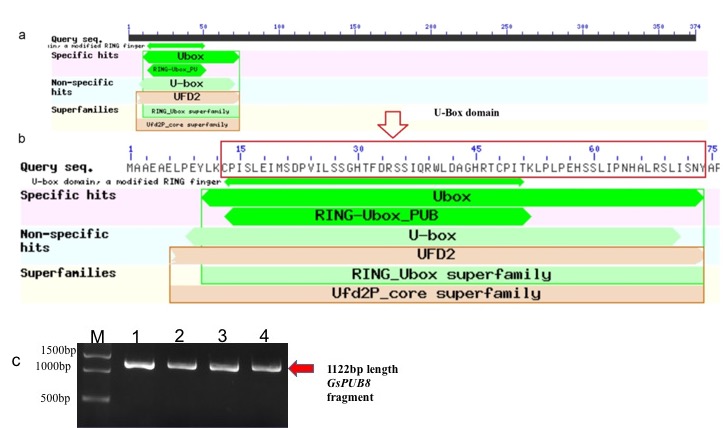


Fig.1 The conserved domain analysis of the GsPUB8 protein (a) A modified RING finger U-box domain (10-74) amino acid residues is shown in green (b) All the identified conserved domains, zoomed in to amino acid residue level (c) PCR product of CDS of PUB8 by gene specific primers, 1122 bp was determined. M: Ladder 1000kb, 2: PCR product.
